# It Constitutes Itself: Health and Community

**Published:** 2006-06-15

**Authors:** Lynne S. Wilcox

Alexis de Tocqueville, a French political scientist and historian who visited America in 1831, is quoted frequently on the distinctions he found between the New and Old Worlds. But some of his other comments highlighted similarities across civilizations: "The village or township is the only association which is so perfectly natural that, wherever a number of men are collected, it seems to constitute itself" (1).

The village, the township, the community — they affect the well-being of their citizens. Since its inception, *Preventing Chronic Disease (PCD)* has published reports of health programs in communities. The Community Voices section in this issue of *PCD* emphasizes communities from the Racial and Ethnic Approaches to Community Health (REACH 2010) program, which was designed to close the gap by 2010 between the best health outcomes and those of minority populations (2). We thank Leandris Liburd of the Division of Adult and Community Health, National Center for Chronic Disease Prevention and Health Promotion, Centers for Disease Control and Prevention, for her efforts in organizing the REACH 2010 article series. Liburd et al describe REACH 2010 (3) and acknowledge the community voices from Portland, Ore (4); Cherokee, NC (5); and Hillsborough County, New Hampshire (6).

Additional community programs are included in our Community Case Studies section. BC Walks is a community-based program designed to encourage moderate-intensity daily walking among 40- to 65-year-old residents of Broome County, New York (7). The quasi-experimental intervention included multimedia ads, community speaker bureaus, and school and worksite walking programs. BC Walks was intended to scale up from a pilot study, and the authors recommend more detailed examination of how the elements of successful campaigns can be duplicated in other settings.

A *community* was originally conceived as a defined geographic area and its residents. We have come to recognize that other forms of villages also have the potential to improve people's health. Several of these alternative community opportunities are also described in this issue. Polacsek et al evaluated the Move and Improve (8) program for worksite wellness in Maine. This program was established by the Eastern Maine Medical Center to encourage physical activity through activity logs, walking clubs, exercise programs, classes, and other community-based activities. The evaluation identified a marked increase in physical activity for program participants, as well as greater weight loss, better diet, and decreased television viewing. The authors recognized serious limitations in the evaluation due to low response rates, cross-sectional study design, and other concerns. Nevertheless, they found important benefits in using a logic model and community-based participatory research.

The A New DAWN (Diabetes Awareness & Wellness Network) Program examined a church-based intervention to educate African American parishioners on how to control type 2 diabetes (9). Methods and baseline measurements of this diabetes self-management instruction series are provided in the Samuel-Hodge et al report; we will look forward to the assessment of its effectiveness. The *`Ohana* Day Project was developed for health and resource education among rural Native Hawaiians. Few communities in the United States could be more challenging to reach, but Gellert et al of the *`Imi Hale* Native Hawaiian Cancer Network were able to provide a 1-day community celebration that provided cancer screening, education, and referral services, with 100% of abnormal findings treated by 6-month follow-up (10).

These are exciting results, and communities have the political will to accomplish even more. De Tocqueville also said of villages:

. . .although the existence of the township is coeval with that of man, its freedom is an infrequent and fragile thing. . . . [Townships] cannot defend themselves with success unless they are identified with the customs of the nation and supported by public opinion (1).

Communities need broad support to constitute themselves and protect their citizens from poor health. Public health plays a critical role in providing that support.
